# Effect of Blade Geometry on γ′ Lattice Parameter and Primary Orientation of SX Cored Turbine Blades (II)

**DOI:** 10.3390/ma16134892

**Published:** 2023-07-07

**Authors:** Jacek Krawczyk, Włodzimierz Bogdanowicz, Jan Sieniawski

**Affiliations:** 1 Institute of Materials Engineering, University of Silesia in Katowice, 1a 75 Pułku Piechoty St., 41-500 Chorzów, Poland; wlodzimierz.bogdanowicz@us.edu.pl; 2 Department of Materials Science, Rzeszów University of Technology, 2 W. Pola St., 35-959 Rzeszów, Poland; jansien@prz.edu.pl

**Keywords:** nickel-based superalloy, single-crystalline blades, cored turbine blades, γ′ lattice parameter, primary orientation, residual stress

## Abstract

The distributions of the lattice parameter of the γ′-phase (aγ′) and angular components of the primary crystal orientation along the lines parallel to the main axis of the single-crystalline CMSX 4-cored turbine blades were studied. The studies were carried out on the regions of the blades located far from the selector and its continuer extension (CE), positioned asymmetrically relative to the blade’s axis. It was found that, similarly to the regions of the blade located close to the CE (studied in part I), at the level of the blade related to the change of its cross-section, there were correlated local changes in aγ′ and the angular components of the primary crystal orientation representing the bending of the dendrites. However, the correlation was less clear due to the presence of low-angle boundaries (LABs) and the intensification of the consequences of the “fanning effect” in the regions far from the CE. It was found that the range of local changes in aγ′ and the angular components of the primary crystal orientation of the blade regions were influenced by both the distance from the CE and the separation of these regions from the CE by surfaces of the cooling bores. It was found that the deviation angle in the [001] direction from the blade axis increased with an increase in the distance from the CE. Based on the aγ′ changes, differences in the alloying element concentration near the cooling bores were discussed.

## 1. Introduction

The turbine blades applied as the component of the turbine hot section of jet engines are required to resist high thermomechanical loads and corrosion caused by an aggressive environment in the combustion chamber. The blades are made as single-crystalline (SX) casts of heat and creep-resistant materials, for example, commonly used superalloys. They have an impressive combination of high-temperature strength, phase stability, and high-temperature oxidation resistance [1,2,3,4]. The most recently used representative is CMSX-4, the second-generation superalloy based on Ni_3_Al, provided by Cannon-Muskegon.

**Figure 1:**
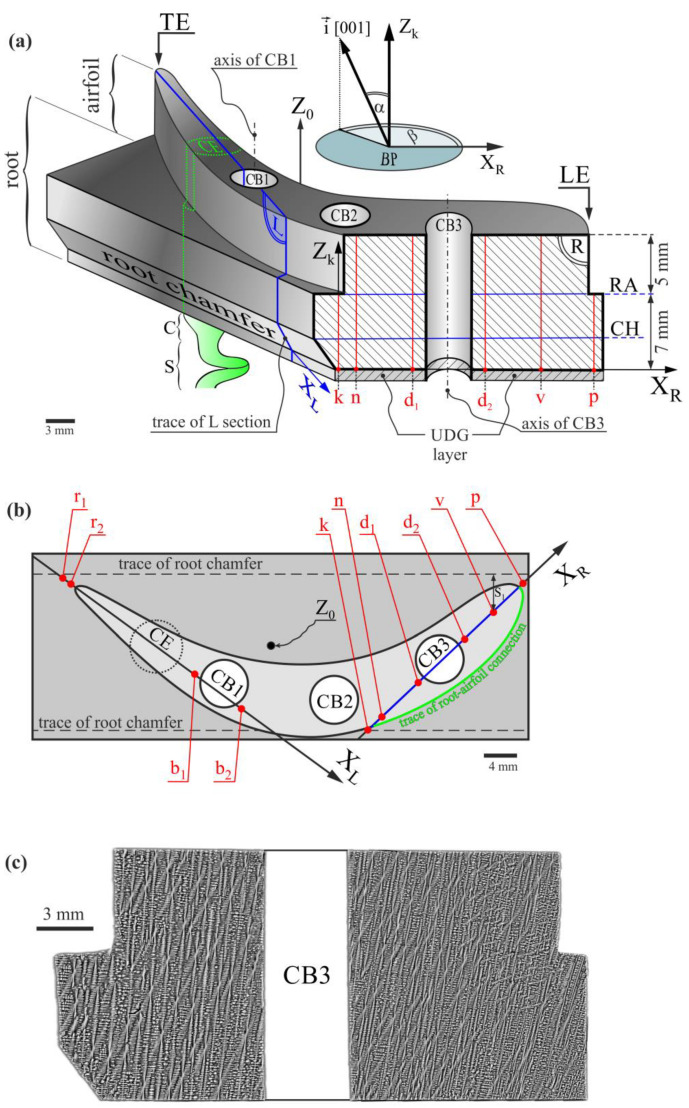
The shape of a model blade with a prepared R-section in oblique view (**a**) and top view (**b**), and the dendritic structure of the R-section (SEM, BSE) (**c**). The insert on the top of Figure 1a shows the scheme of the α and β angles definition for measuring line k, where Z_k_ is its axis. The axes of the other measuring lines have not been marked for the figure’s clarity. Z_0_—main vertical axis of the blade; CH—chamfer level; RA—root–airfoil connection level; k, n, d_1_, d_2_, v, p—measuring lines; LE, TE—leading and trailing edges of the airfoil, respectively; S—spiral selector; C—a continuer of the selector; CE—continuer extension; CB1, CB2, CB3—cooling bores; UDG layer—unsteady dendrite growth layer. A description of other symbols is provided in the text.

Single-crystalline turbine blades may be produced during the process of directional dendritic crystallization in ceramic molds using the Bridgman technique. The blades are often produced with channels and bores through which cooling air flows during engine operation. They are called cored blades because the casting molds use ceramic cores to form the cooling bores. The crystallization by the Bridgman method allows for the obtaining of blades with the required crystal orientation of [001]-type, which gives the blade a high creep resistance [1]. In practice, such an orientation means that the [001]-type crystal direction is nearly parallel to the main blade axis Z_0_ (Figure 1a). Hence, the α angle between [001] and Z_0,_ characterizing the primary crystal orientation, is low. The [001]-type crystal direction is parallel to the γ-dendrite growth direction in the crystallization of blades by the Bridgman method. Stochastic misorientation of the neighboring dendrites, and thus the areas of the blades, and also the misorientation created due to the complex shape of the blade [5] can allow the formation of low-angle boundaries (LABs) [6,7,8] that reduce the strength parameters of the blade. The crystal orientation of SX blades significantly influences thermal fatigue strength [9], and its local changes may also affect the corrosion resistance of SX Ni-base superalloys [10]. Local changes in the crystal orientation in the blade are expressed by local changes to the α angle during the formation of the γ-dendrites that grow in the casting mold. Such dendritic structure inhomogeneity may also cause the creation of sliver defects, which influences the mechanical properties of the blades [11]. Therefore, it may be concluded that the local change of the primary crystal orientation causes several types of defects during crystallization due to dendrite growth disturbance. A large concentration of defects related to the local crystal misorientation, such as low-angle boundaries, may reduce the blade’s high-temperature strength and creep resistance. Hence, it is necessary to analyze, in the blades, the spatial distribution of the primary crystal orientation mainly characterized by the α angle that describes the inclination of the [001] crystal direction to the main blade axis Z_0_ or axes parallel to it. The full primary orientation of the local blade areas is additionally described by the rotation of the [001] direction around axes parallel to Z_0_, represented by the β angle (Figure 1). Therefore, the spatial distribution of the β component of the primary orientation can also be important.

Local bending of single-crystalline dendrites during solidification may be related to the crystal misorientation of the neighboring blade areas and, thus, the formation of macroscopic subgrains and LABs [12,13,14]. The bending of dendrites causes the non-parallel growth of adjacent dendrites, generally in small crystal lattice disorientation. Several reasons exist for dendrite bending during growth, morphological and mechanical bending being notable factors [15]. Morphological bending is related to the local changes in the chemical composition of the liquid, influencing the growth direction of the dendrites without changing their crystal orientation. Some reasons for the changes in chemical composition are similar to those in the case of the so-called dendritic segregation of alloying elements that superalloys contain a lot of [16,17,18,19,20]. Moreover, if there is a change in the crystal orientation of the growing dendrite, it means there is a mechanical bending related to the local distortion of its crystal lattice. Both types of bending cause a local change in the lattice parameter and, thus, the formation of residual stresses, adversely affecting the stability of protective coatings applied to the surface of the blades at subsequent stages of their production [21,22,23,24]. The reason for the dendrites’ local bending may be the casting mold’s complex shape that gives the required shape to the single-crystalline blades. In turn, the bends may be related to a change in chemical composition. Characterization of single-crystalline blades for heterogeneity and the determination of the causes for such heterogeneity formation are essential because they can determine the ways of eliminating or reducing such heterogeneity.

The engines run at ever-higher turbine inlet temperatures to meet ever-increasing operating demands. The blades of the hot turbine work in temperatures close to those at the start of the melting process of the superalloy they are made of. The blades are cooled by the air passing through the inner bores of complex geometry, reducing their temperature. A more complicated cooling is based on “blade film cooling”, based on the supply of cold air through the channels to the blade’s surface which eliminates direct contact with the gas flowing out of the combustion chamber to a certain extent. In any case, cooling bores must be created within the blades approximately parallel to the main blade axis Z_0_ to supply air from the root to the airfoil. The blades with cooling bores are produced using a casting mold prepared with internal ceramic cores [25,26,27,28].

Directional crystallization of the blades using the Bridgman method is based on the growth of single-crystalline dendrites of the γ-phase toward the [001] crystal direction. During crystallization, when the temperature is decreased below the solvus, the γ → γ′ + γ_II_ transition takes place, where γ′ is a solid solution of the ordered cubic Ni_3_Al phase with numerous alloying elements like W, Re, Ta, Ti, and γ and γ_II_ that are the disordered cubic phases of the Ni-based solid solution. However, this phase transition does not change the crystal orientation. It was assumed that the spatial distribution of the crystal orientation given during the growth process of the γ-dendrites is inherited by the distribution of the γ′-phase orientation. On the other hand, local changes in the lattice parameter a_γ′_ of this phase will be located in the same areas of the blade where changes in the lattice parameter of the γ-phase exist before the transformation. Therefore, the analysis of the spatial distributions of the primary crystal orientation of the γ′-phase and its lattice parameter, and the correlation between them in the blades in an as-cast state, can be related to the γ-dendrite growth process. The measurement of these distributions can be performed quite easily in the CMSX-4 superalloys using X-ray diffraction methods. It is justified because the volume fraction of the γ′-phase is above 70% in the as-cast superalloy.

In the first part of the studies reported in [5], the distribution of the α angle and the a_γ′_ lattice parameter along the measuring lines traced parallel to the main axis Z_0_ of the blade on the surface of the L-section (Figure 1a,b) that passed through the selector’s continuer extension (CE) region was examined. The measuring lines were located relatively close to the CE region (Figure 1b). Since, at a certain stage of Bridgman crystallization, the dendrites’ growth area and the crystallization front enlarges from a narrow selector or its continuer to a wide root, it is therefore important to compare the distributions of α(Z) and a_γ′_(Z) (where Z is parallel to Z_0_) measured for the L-section surface, i.e., close to the selector or continuer (Figure 1a,b) with similar distributions measured for the R-section surface, i.e., far from them. It is especially important for bulk blades with large cross-sections.

As presented in [5], clear changes in the relations of α and a_γ′_ as the functions of coordinates of the measuring line axes traced parallel to Z_0_ on the surface of the L-section were found on certain levels, i.e., for certain Z. These changes were related to chamfer of the root surface and the root–airfoil connection; in other words, they occurred at the CH and RA levels (Figure 1a). It was found that there are significant differences in the α and a_γ′_ distributions along the lines traced at different areas of the L-section. This was the case for the measuring lines that matched with the areas of the blade root over which the airfoil is not located and areas in which the airfoil is a continuation of the root, e.g., the area where the k- and n-type lines are traced in Figure 1a. In addition, it was found that the α and a_γ′_ distributions along the n-type lines traced near the first cooling bore (CB1) have specific characteristics. Therefore, in the currently presented studies, areas of the same type were analyzed but located in the R-section at a much longer distance from the selector and its continuer extension, considering the top view.

Although the problem of dendrite bending illustrated by α changes and combined with residual stresses has been the subject of many studies [24,29,30], none present a precisely defined bending angle nor relate it to the geometry of the blades.

The goal of the current study was to analyze the influence of the external and internal surfaces of single-crystalline cored turbine blades made of CMSX-4 superalloy on changes of the γ′ lattice parameter (a_γ′_) and angular components α and β of the primary crystal orientation of the regions located far from the selector, considering the top view, on the R-section. The influence was examined based on changes in the a_γ′_ and α and β angles occurring at the levels of the root chamfer and the root–airfoil connection for the measuring lines traced near the external surface of the blade and the internal surface of its cooling bores. The areas of the blade root over which the airfoil is not located and areas in which the airfoil is a continuation of the root, as well as the areas similar to the latter but located near the walls of the cooling bore, were examined. The current paper describes the continuation of the studies presented in [5] in which the research results on regions of the L-section located close to the selector and its continuer extension were presented.

## 2. Material and Methods

The castings were obtained using CMSX-4 superalloy in the Research and Development Laboratory for Aerospace Materials, Rzeszów University of Technology, Rzeszów, Poland. The directional crystallization of the Bridgman method was applied using the ALD Vacuum Technologies Co. (Hanau, Germany) VIMIC 2E–DS/SC industrial furnace. The withdrawal rate was 3 mm/min. The nominal chemical composition of CMSX-4 melt was: 5.6 Al, 1.0 Ti, 6.5 Ta, 6.5 Cr, 0.6 Mo, 6.0 W, 9.0 Co, 3.0 Re, 0.1 Hf, less than 0.002 C, Ni bal. (wt.%). The crystallization process was conducted in the ceramic mold with a starting temperature of 1520 °C.

The blade castings contained three cylindrical cooling bores, CB1, CB2, and CB3, and a spiral selector (S) with the continuer (C). The selector was asymmetrically located relative to the main blade axis Z_0_ (Figure 1a,b). The continuer extension (CE) marked in Figure 1a,b was a cylindrical shape bounded by the projection of the continuer’s perimeter into the blade. The model blades with a relatively short airfoil, 5 mm long (Figure 1a), with cooling bores of the simplest cross-section shape—circular—and the simplest orientation—parallel to the main blade axis Z_0_—were used for the tests.

The model blades were cut along the plane, which was parallel to the main axis Z_0_ and passed through the airfoil’s leading edge (LE) and the center of the third cooling bore (CB3), located farthest from the CE (Figure 1a). The longitudinal R-section of the shape presented in Figure 1a was prepared for the measurements using the standard metallographic procedure [31].

The dendritic structure of the analyzed section surface was visualized with the use of a JSM-6480 JEOL SEM microscope (JEOL Ltd., Tokyo, Japan). Figure 1c shows an image of the dendritic structure revealed on the surface of the R-section. The image was obtained using backscattered electron (BSE) imaging by collecting numerous smaller images.

The measurements of the γ′ lattice parameter (a_γ′_) and the angular components of the primary orientation (α and β angles) have proceeded in the Research and Development Laboratory for Aerospace Materials, Rzeszów University of Technology, Rzeszów, Poland. The specialized EFG Freiberg Instruments X-ray diffractometer (Freiberg Instruments, Freiberg, Germany) [32] was used during tests. The pseudo-parallel primary X-ray beam was 0.8 mm in diameter. The full primary crystal orientation (orientation of primary dendrite arms) is defined by two angles, α and β, which describe the tilt and rotation of the primary dendrite arms growing along the [001] unit vector $\vec{i}$, presented in the insert of Figure 1a. A laser scanner mapped out the measuring lines parallel to the blade axis Z_0_ on the R-section surface. The a_γ′_, α, and β values were calculated with the software equipment of the diffractometer, based on the Ω-scan method [32]. The mean error of the a_γ′_ and α and β measurements was 0.0005 Å and 0.006°, respectively.

The a_γ′_, α, and β measurements were completed at points along several lines traced parallel to the main blade axis Z_0_ on the R-section surface. The chosen lines are as follows: k, located near the side surface of the root; n, located near the outside surface of the airfoil; d_1_ and d_2_, located nearby the internal surfaces of the blade on the left and right sides of the CB3, respectively; v and p, located close to the LE of the airfoil (Figure 1a,b). Lines p and k cover only the blade root because the airfoil is not located directly above, and the other lines cover the root together with the airfoil because, for them, the airfoil is a continuation of the root.

## 3. Results and Discussion

**Figure 2:**
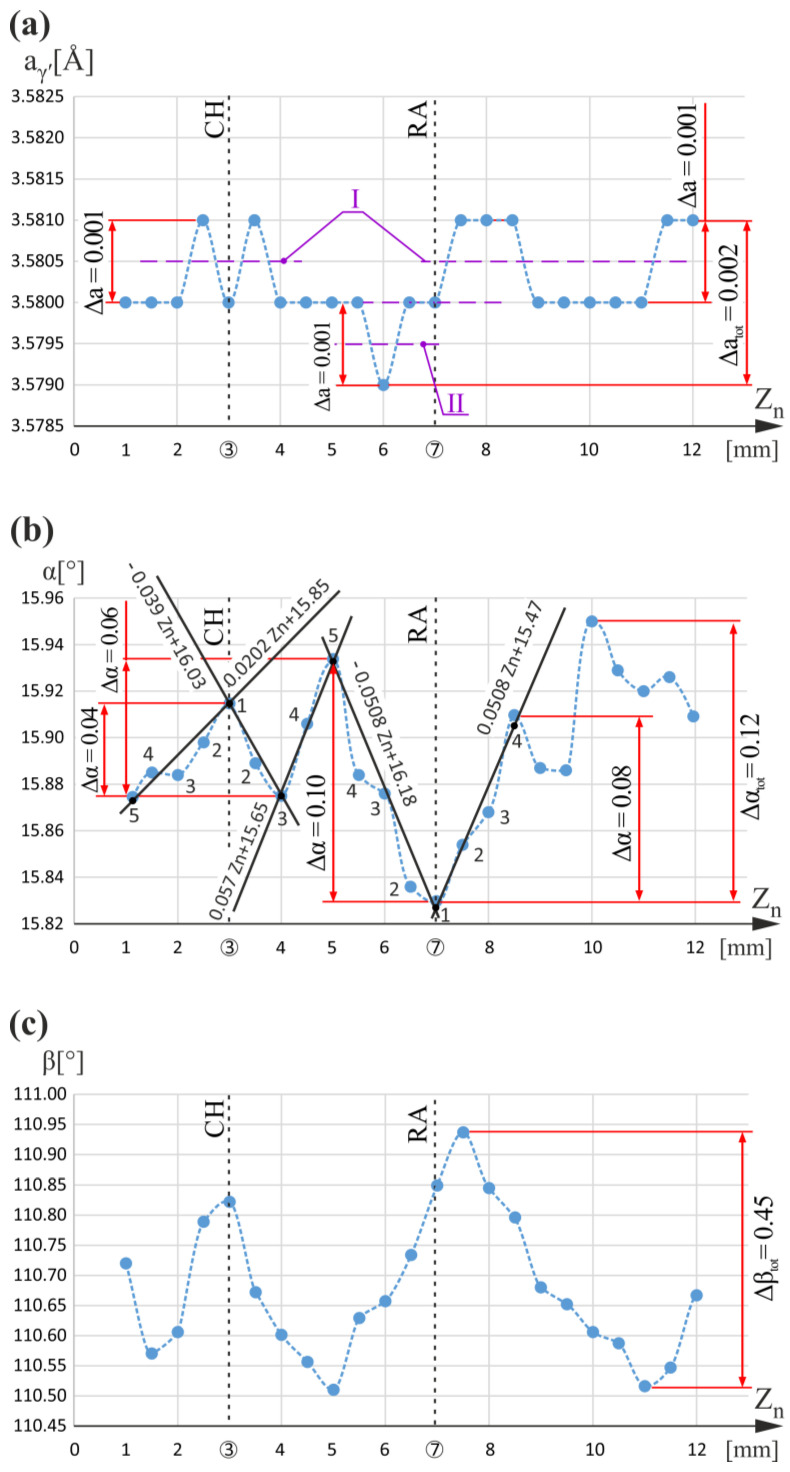
Graphs of the a_γ′_(Z_n_) (**a**), α(Z_n_) (**b**), and β(Z_n_) (**c**) relationships obtained for measuring line n. Z_n_ is parallel to Z_0_. The conditions Z_n_ = 3 mm and Z_n_ = 7 mm determine the level of root chamfering (CH) and root–airfoil connection (RA), respectively.

Figure 2 shows the graphs of the a_γ′_(Z_n_), α(Z_n_), and β(Z_n_) relationship obtained for the measuring line n. The value of a_γ′_ for the Z_n_ ranged between 1 mm and 5.5 mm and varied about 3.5805 Å in the range of 0.001 Å, which corresponds to the fluctuations of a_γ′_ in an undisturbed dendritic structure [5,33]. However, for Z_n_ equal to 6 mm, a_γ′_ decreases, and then, near Z_n_ equal to 7 mm corresponding to the RA level, it increases by 0.002 Å, reaching the value of 3.5810 Å. The total range of a_γ′_ changes for the entire Z_n_ measurement range equal to 0.002 Å and was marked as Δa_tot_ (right side of Figure 2a). On the graph of the α(Z_n_) relationship, there is a minimum for Z_n_ equal to 7 mm. The difference in the slope coefficients of local linear approximations (the trend lines) of α(Z_n_) for Z_n_ less than 7 mm (measuring points 1–5) and Z_n_ greater than 7 mm (measuring points 1–4) is 0.1016°/mm (0.0508 − (−0.0508) = 0.1016 (°/mm)). The change in α near Z_n_ equal to 7 mm varies in the range from 0.08° to 0.10°. The details of the extremes analysis of the α(Z)-type relationships in the context of its correlation with α_γ′_(Z) changes induced by dendrite bending were presented for the first time in [5]. Additionally to the extreme at Z_n_ equal to 7 mm on the α(Z_n_) graphs, there are three more extremes: near CH at Z_n_ equal to 3 mm and at Z_n_ equal to 4 mm and 5 mm. However, both the value of the slope coefficients difference and the α value changes were the highest for the minimum at Z_n_ equal to 7 mm. Nearby Z_n_ of 3 mm value, where the maximum α(Z_n_) is observed, the α changes equal to 0.04° are much smaller, both for Z_n_ less than 3 mm (measuring points 1–5) and for Z_n_ greater than 3 mm (measuring points 1–3), and the difference in the slope coefficients of the α(Z_n_) trend lines is 0.0592°/mm (0.0202 − (−0.039) = 0.0592 (°/mm)). These two parameters are smaller than that for a minimum at Z_n_ equal to 7 mm. For the minimum at Z_n_ equal to 4 mm, the difference in the slope coefficients of α(Z_n_) is 0.0897°/mm (0.0507 − (−0.039) = 0.0897 (°/mm)), which is lower than that determined near Z_n_ equal to 7 mm. Additionally, the change of the α value is equal to 0.04° for Z_n_ less than 4 mm (measuring points 1–3) and equal to 0.06° for Z_n_ greater than 4 mm (measuring points 3–5), and is also much smaller than that for Z_n_ near 7 mm.

For maximum at Z_n_ equal to 5 mm, the difference in the slope coefficients α(Z_n_) is 0.1078°/mm (0.057 − (−0.0508) = 0.1078 (°/mm)), which is comparable to that determined for the minimum at Z_n_ equal to 7 mm. A change in the α value of 0.10° for the Z_n_ ranged between 5 mm and 7 mm is the same for both extremes at Z_n_ equal to 5 mm and 7 mm. However, changes in the α values of 0.06° for Z_n_ less than 5 mm (measuring points 3–5) are smaller than those for Z_n_ greater than 7 mm, equal to 0.08° (measuring points 1–4). As explained in [5], the extremes of α(Z_n_) correspond to the bends of the dendrites. Since the degree of dendrite bending is determined both by the value of the α changes and the difference of the slope coefficients, therefore, it should be assumed that the bending of the dendrites at the levels of Z_n_ equal to 3 mm (CH level) and Z_n_ equal to 4 mm, expressed by the extremes of α(Z_n_), was insufficient to change the lattice parameter α_γ′_ by more than the fluctuation range (0.001 Å). However, the degree of dendrite bending at Z_n_ equal to 5 mm, represented by maximum α(Z_n_), and at Z_n_ equal to 7 mm, represented by minimum α(Z_n_), was so significant that it caused a decrease in α_γ′_ from level I to level II of the fluctuation at Z_n_ equal to 6 mm, and an increase from level II to level I of the fluctuation above Z_n_ equal to 7 mm, respectively.

For Z_n_ greater than 8 mm, the graph of the α_γ′_(Z_n_) relationship (Figure 2a) only shows a fluctuation range of 0.001 Å about level I. This means that the growth of the dendrites in this area was undisturbed despite the changes of α(Z_n_), and the dendrite bends may have been created after the crystallization. The α(Z_n_) changes may also be related to the vicinity of the measuring line n to the walls of the CB2. A much clearer correlation between α_γ′_ changes and α(Z) extremes occurs for the regions of the blades located near the CE, as described in [5]. This is because for these regions, in wide ranges of Z_n_, the fluctuation areas, with 0.001 Å serving as reference, occurred. Unfortunately, among the R-section measuring lines located far from the CE, the widest range of α_γ′_ fluctuation occurred for line n. To explain these incomprehensible effects, it was decided to perform additional measurements of the β-angle component of the primary orientation. The additional reasons will be described afterward. The β component expresses the rotation of the “i” [001] unit vector about the Z_n_ axis (Figure 1a, insert), which is parallel to the main axis Z_0_ of the blade. The “i” unit vector is consistent with the dendrites’ growth direction. In other words, β describes the rotation of the dendrites around some axis (e.g., axis Z_n_ or Z_k_) that is parallel to Z_0_.

A comparison of the α(Z_n_) and β(Z_n_) relationship graphs (Figure 2b,c) shows the correlation between local extremes at points with coordinates Z_n_ equal to 3 mm, Z_n_ equal to 5 mm, and near the point with coordinate Z_n_ equal to 7 mm. This means that the tilting of the dendrites from the Z_n_ axis (change in α value) and their rotation around this axis (change in β value) occurs simultaneously during growth. However, for the entire Z_n_ measurement range, the total change in the rotation angle, marked on the right of Figure 2c as Δβ_tot_ and equal to 0.45°, was significantly higher than the change in the tilt angle marked on the right of Figure 2b as Δα_tot_ was equal to only 0.12°.

To be able to reliably isolate any local a_γ′_ changes greater than the stochastic fluctuations of 0.001 Å (for example, related to the CH or RA levels) on any graph of a α(Z)-type relation, there must be a noticeably long (for a relatively long Z range) segment on which only a_γ′_ fluctuations of 0.001 Å occur. Such types of segments serve as reference areas. For the entire R-section, the graph in which these segments are clearly visible occurred only for the measuring line n (Figure 1a). Local a_γ′_ changes above 0.001 Å are related to any disturbances in the formation of the dendrite array visualized on the blades’ longitudinal sections. In [5], it was shown that CE can serve as an example of an undisturbed region. The disturbances are formed mainly during the growth of dendrites and may be related to the shape of the blade and/or the shape of the casting mold.

**Figure 3:**
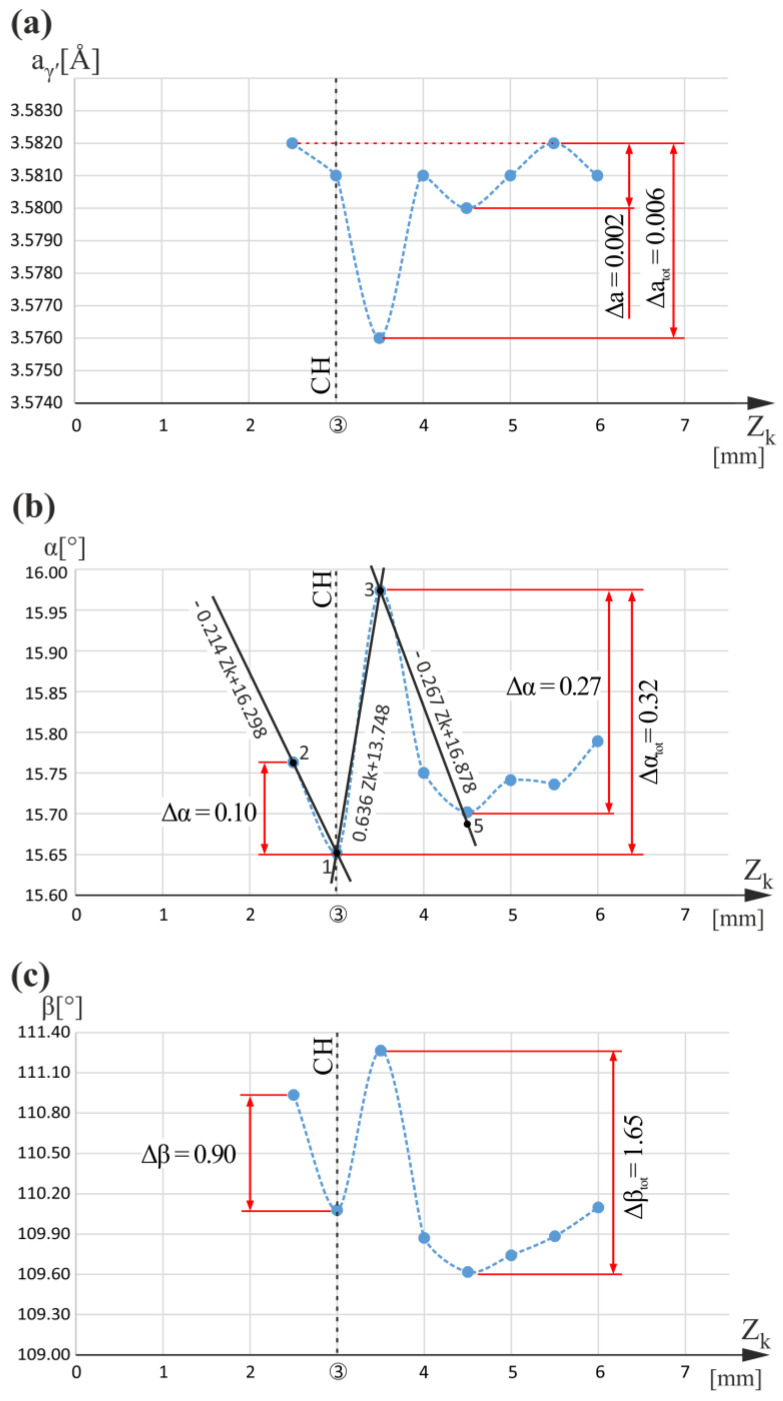
Graphs of the a_γ′_(Z_k_) (**a**), α(Z_k_) (**b**), and β(Z_k_) (**c**) relationships obtained for measuring line k. The condition Z_k_ = 3 mm determines the root chamfering (CH) level.

Figure 3 shows graphs of the a_γ′_(Z_k_), α(Z_k_), and β(Z_k_) relationships obtained for the measuring line k (Figure 1a,b). The airfoil is not located over the root for line k. Slightly higher than the CH level, for Z_k_ equal to 3.5 mm, there is a clear minimum of the a_γ′_(Z_k_). One of the effects of this minimum is that when Z_k_ decreases below 3.5 mm, there is a large a_γ′_ increase of 0.006 Å. An effect of similar character is also observed and described in [5] during the analysis of the root measuring lines r_1_ and r_2_ of the L-section, the location of which is marked in Figure 1b. However, the increase in a_γ′_ value was much smaller (0.002 Å) and occurred slightly lower than the CH level. The r_1_ and r_2_ measuring lines are positioned farther from the cooling bores, whereas line k is near the CB2. Therefore, it was assumed that for the areas of the root located near the cooling bore and over which the airfoil is not situated, changes in a_γ′_ are much greater. This assumption was confirmed by the small changes of a_γ′_ (0.002 Å) found for line p (see Figure 1a,b) positioned farther from the CB3 (see below). Since the a_γ′_ depends on the concentration of alloying elements, it can be concluded that local changes in the chemical composition related to the root chamfer are much higher near the cooling bores.

The graph in Figure 3a shows that between Z_k_ of 4.5 mm and 5.5 mm, there is an increase in a_γ′_ of 0.002 Å. This change is difficult to explain and perhaps may be related to the closeness of the measuring line k to two fragments of the casting mold wall simultaneously: horizontal, limiting the root, and vertical, limiting the airfoil (see Figure 1a). Such surface refraction of the casting mold surface may cause the generation of significant residual stresses. They probably precluded the measurement of a_γ′_ for Z_k_ greater than 6 mm because the X-ray diffraction conditions were not met, and no diffraction reflexes were obtained. Additionally, for Z_k_ less than 2.5 mm, diffraction did not occur, probably for a similar reason because there is a refraction of the mold surface related to the chamfer of the root (Figure 1a).

Figure 3b presents the graph of the α(Z_k_) relationship. The figure analysis allows us to conclude that a minimum of the α(Z_k_) for Z_k_ equal to 3 mm (at the CH level), and the maximum of α(Z_k_) for Z_k_ equal to 3.5 mm were observed. The changes of α recorded nearby the minimum are 0.1° (measuring points 1–2) and 0.32° (measuring points 1–3), respectively. The value of 0.32° is the largest for the entire Z_k_ measuring range and is therefore marked as Δα_tot_. The changes in the slope coefficients of the α(Z_k_) trend lines, for the minimum and the maximum, were 0.850°/mm (0.636− (−0.214) = 0.850 (°/mm)) and 0.903°/mm (0.636 − (−0.267) = 0.903 (°/mm)) and were much larger than for line n (Figure 2b). This means that there was a significant local bending of dendrites at the CH level and a slightly higher bending at Z_n_ equal to 3.5 mm, much greater than that observed for line n.

The β(Z_k_) relationship graph is presented in Figure 3c. On the CH level and above it, local changes in β have a character similar to that in α, and are correlated with α changes. However, the scope of local β changes is much wider. The observed decrease in β at Z_k_ equal to 3 mm (the CH level) is 0.90°, and in α it is only 0.10°. For the entire measurement range of Z_k_, the total β change, marked as Δβ_tot_, is 1.65°, and a similar total α change Δα_tot_ is 0.32°. From the correlation and the fact that local changes in the β angle are generally higher than changes in the α angle, it follows that during the growth of the dendrites, their rotation rate relative to axis Z_k_ parallel to main blade axis Z_0_ was significantly higher than their tilting rate relative to these axes.

The rates of the change in α angle, determined by the absolute value of the slope coefficients of the trend line, are linear approximation of α(Z_k_), for Z_k_ less than 3 mm and in the range between 3 mm and 3.5 mm, as well as, in the range between 3.5 mm and 4.5 mm are 0.214°/mm, 0.636°/mm, and 0.267°/mm, respectively (Figure 3b). The difference of these coefficients on the right and left of Z_k_ equal to 3 mm is 0.750°/mm (0.636 − (−0.214) = 0.750 (°/mm)), and on the left and right of Z_k_ equal to 3.5 mm are 0.903°/mm (0.636 − (−0.267) = 0.903 (°/mm)). These differences are some of the parameters that determine the degree of dendrite bending at the local extremes in α(Z_k_) [5], and they are similar for the extremes of Z_k_ equal to 3 mm and 3.5 mm. Additionally, the degree of dendrite bending depends on the absolute value of changes in α near the extremes. For the extreme at Z_k_ equal to 3.5 mm, on their right side, the α change is 0.27° (Figure 3b), and on the left side, it is 0.32°, which is equal to Δα_tot_. On the right side of the extreme for Z_k_ equal to 3 mm, the change in the α angle was equal to 0.32°, which is equal to Δα_tot_. In contrast, on the left side, it was not possible to precisely determine this change because, for Z_k_ less than 2.5 mm, the measurement of the α angle, as well as the β angle and the a_γ′_ lattice parameter, failed. The reason may be that the diffraction conditions in this range of Z_k_ were not satisfied because of the high residual stress in the vicinity of both external and internal (CB2, Figure 1b) mold walls. Therefore, it cannot be determined whether large changes in a_γ′_ near Z_k_ equal to 3.5 mm (Figure 3a) may be related to the minimum in α(Z_k_) at Z_k_ equal to 3 mm or the maximum in α(Z_k_) at Z_k_ equal to 3.5 mm. It was estimated and assumed that to the left of Z_k_ equal to 3 mm, the change in α is 0.10° and the change in β is 0.90° (Figure 3b,c).

**Figure 4:**
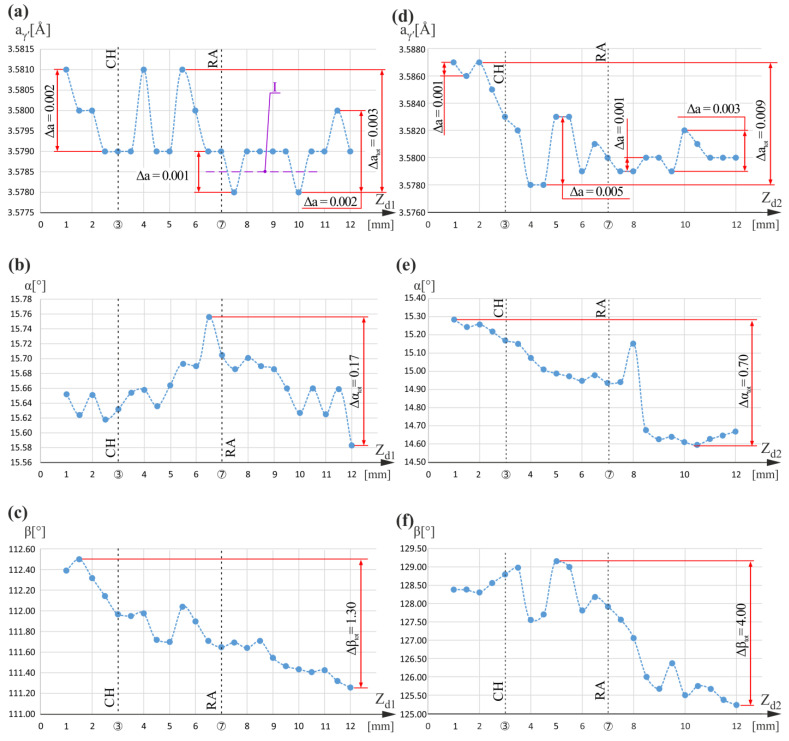
Graphs of the a_γ′_(Z_d1_) (**a**), α(Z_d1_) (**b**), and β(Z_d1_) (**c**) relationships obtained for measuring line d_1_; and graphs of the a_γ′_(Z_d2_) (**d**) α(Z_d2_) (**e**) and β(Z_d2_) (**f**) relationships obtained for measuring line d_2_. Z_d1_ and Z_d2_ are parallel to Z_0_ and located near at left and right sides of CB3.

Figure 4 presents graphs of the a_γ′_(Z_d1_), α(Z_d1_), and β(Z_d1_) relationships for measuring line d_1_, as well as graphs of the a_γ′_(Z_d2_), α(Z_d2_), and β(Z_d2_) relationships obtained for measuring line d_2_. Measuring lines d_1_ and d_2_ were located near CB3 (Figure 1a,b) on its left and right side. The a_γ′_(Z_d1_) graph has some characteristics similar to those shown in Figure 2 as well as to those presented in [5] for the measuring lines located nearby CB1 (measuring lines b_1_ and b_2_ in Figure 1b). In the graph from Figure 4a, some level I a_γ′_ changes in a range of 0.001 Å can be distinguished. These changes correspond to an undisturbed dendritic structure without significant bending of the dendrites [5,33] and, therefore, can be called fluctuations. Comparing the value of Z_d1_ equal to 6.5 mm, for which the fluctuation starts with the coordinates of changes occurring in the α(Z_d1_) graphs (Figure 4b), a correlation of the α maximum at Z_d1_ equal to 6.5 mm with a decrease in a_γ′_(Z_d1_) greater than the fluctuation range near Z_d1_ equal to 6.5 mm can be observed. It confirms the conclusion from [5] that the large local bending of dendrites is related to the local change in a_γ′_ above the fluctuation range of a_γ′_(Z_d1_).

For α(Z_d1_) in the scope limited by Z_d1_ ranged between 1 mm and 6 mm and between 8 mm and 12 mm, no clear correlations between α(Z_d1_) and a_γ′_(Z_d1_) were observed despite changes of a_γ′_ equal to 0.002 Å, that is higher than the fluctuation range for the scope limited by Z_d1_ between 1 mm and 6 mm, and for Z_d1_ greater than 10 mm. It can be assumed that in these areas, measuring line d_1_ passes through macroscopic low-angle boundaries (LAB), near which a_γ′_ changes significantly [34]. Such boundaries, which occurred in large numbers in similar-cored blades near the CB3, were shown in [35]. Because the misorientation angle of the LABs is related to both the α and β component changes, it could be assumed that changes in a_γ′_ may be related not only to the α(Z_d1_) relationship extremes but also to the β(Z_d1_) relationship extremes. To check this assumption, the β(Z_d1_) relationship was experimentally determined, which is shown in Figure 4c. Unfortunately, it is difficult to observe a clear correlation between changes in a_γ′_ and changes in β(Z_d1_). Only in the β(Z_d1_) graph can a general decrease in the β value be observed for the entire range of Z_d1_. It may be related to the so-called “fanning effect” [24] or/and to the directing of growing dendrites by vertical mold walls parallel to the Z_0_ axis, similarly to the case of the so-called “force directing” effect [36,37]. Comparing the α(Z_d1_) and β(Z_d1_) graphs, it can be observed that the total changes in the β value Δβ_tot_ equal to 1.3° are much wider than the changes in the α value Δα_tot_ that is equal to 0.17°.

On the right side of the CB3, the relation of the γ′ phase lattice parameter a_γ′_ and the Z_d2_ is visualized by the graph shown in Figure 4d. In the graph, only in small areas between Z_d2_ equal to 1 mm and 2 mm, and between 7.5 mm and 9.5 mm, are the a_γ′_ fluctuations in the 0.001 Å range observed. In other, much wider areas, the changes in a_γ′_ are much greater and equal to 0.009 Å, 0.005 Å, and 0.003 Å for different ranges of Z_d2_. This means that there is a much greater amount of local changes in chemical composition and higher segregation of alloying elements on the right side of CB3 than on the left, where the total changes of a_γ′_ were only 0.003 Å.

In the α(Z_d2_) graph, against the background of a general decrease in α, only one clear maximum is visible at Z_d2_ equal to 8 mm. It is unrelated to any change on the a_γ′_(Z_d2_) graph greater than the fluctuation range. The β(Z_d2_) relationship graph (Figure 4f) has several extremes of varied character., e.g., for Z_d2_ equal to 3.5 mm, 4.0 mm, and 5.0 mm, as well as for Z_d2_ equal to 9.5 mm. Near these values of Z_d2_ in the a_γ′_(Z_d2_) graph, there are significant changes in a_γ′_. It can be concluded that the change in a_γ′_ can be influenced not only by the bending of dendrites related to the change in the α angle but also by the bending related to the β angle corresponding to the rotation of the dendrites around the axes parallel to the main axis Z_0_ of the blade. Comparing the α(Z_d2_) and β(Z_d2_) graphs, it can be observed that the total change in β defined for the entire measuring range is equal to 4.00° (Δβ_tot_), and is much higher than the total change in α, which is only equal to 0.70° (Δα_tot_). In Figure 4c,e,f, for the entire Z_d1_ and Z_d2_ measuring ranges, there is a tendency to decrease the values of the α and β angles. This may be related to the so-called “fanning effect” described in [24].

The Δa_γ′_ changes in the range from 0.003 to 0.009 Å observed for the d_2_ measuring line are comparable or higher to those shown in [38] for areas near the dendrite core and in the interdendritic regions. The convergent beam electron diffraction (CBED) method was used for the a_γ′_ measurement of the heat-treated CMSX-4 superalloy. In [39], for the same heat-treated superalloy, changes in a_γ′_ values for similar areas were determined by high-resolution X-ray diffraction. The area covered by the primary X-ray beam had a diameter of 0.1 mm, which is eight times smaller than the beam of 0.8 mm in diameter used in our research. In both papers [38,39], fragments of single-crystalline castings of CMSX-4 superalloy obtained by the Bridgman method, with a primary arm spacing (PAS) of about 300 μm, were examined. It can be concluded that the crystallization rate determined by the rate of pulling out of the mold from the high-temperature zone in the Bridgman method applied in the above-presented studies was about 3 mm/min., i.e., the same as the rate of obtaining the blades studied in our research. From the results presented in [39], it can be concluded that the maximum changes in a_γ′_ in the area 100 μm away from the dendrite core compared to the changes in a_γ′_ inside the dendrite core were 0.003 Å.

In our studies, on the longitudinal cross-section of the blades, the distribution of “hourglasses” visualizing the cores and secondary arms of the dendrites was such that it allowed an X-ray beam with a diameter of 0.8 mm to cover, separately, the areas located near the cores of dendrites or areas near the ends of secondary arms. Changes in a_γ′_ in the range of 0.001 Å, which can be called fluctuations due to their stochastic character, correspond to these two locations [33]. In contrast, Δa_γ′_ changes above 0.001 Å are caused by specific local crystallization conditions related to the complex shape of the entire blade [40,41,42,43].

**Figure 5:**
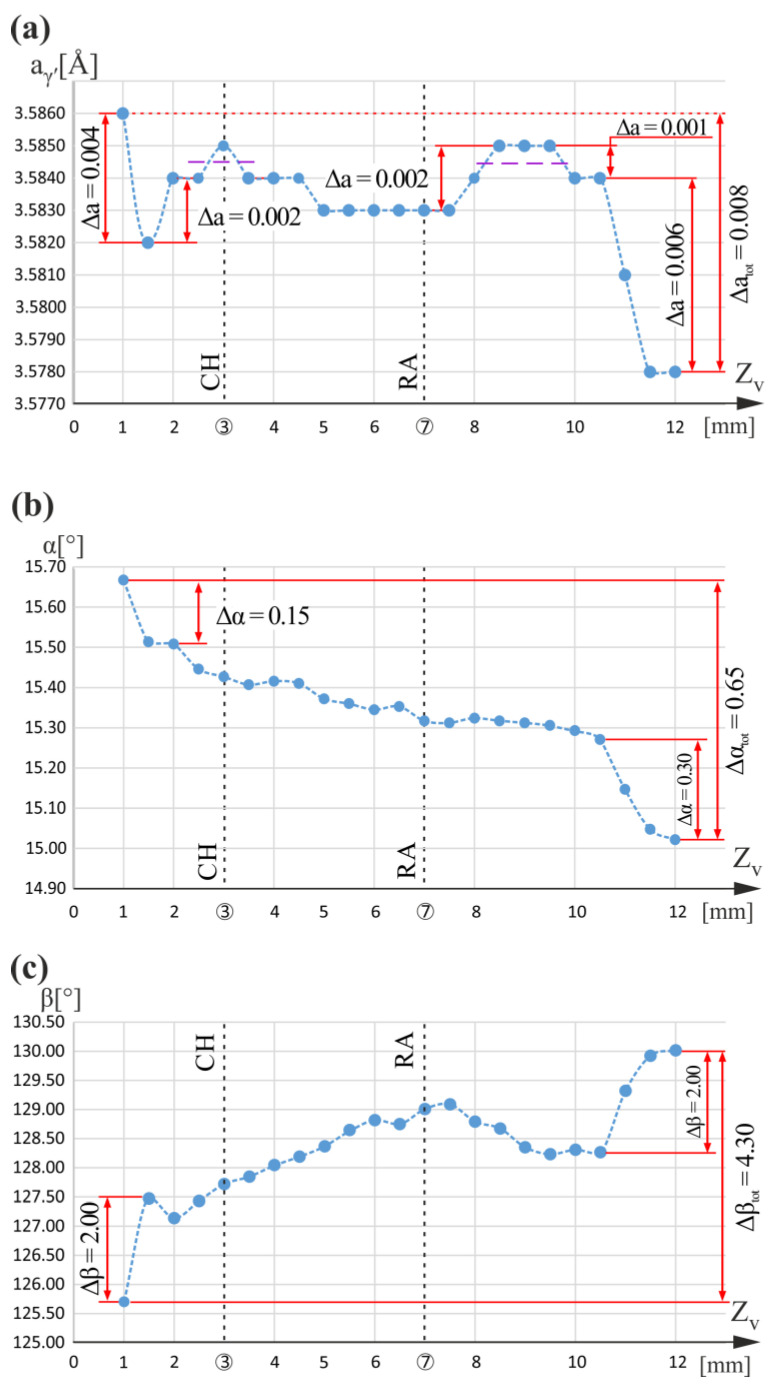
Graphs of the a_γ′_(Z_v_) (**a**), α(Z_v_) (**b**), and β(Z_v_) (**c**) relationships obtained for measuring line v. Z_v_ is parallel to Z_0_.

Figure 5 shows a graph of the a_γ′_(Z_v_), α(Z_v_), and β(Z_v_) relationship obtained for measuring line v (Figure 1a,b). Local fluctuations of 0.001 Å in the a_γ′_ lattice parameter corresponding to the undisturbed dendritic structure occur in the area limited by Z_v_, ranging only between 8 mm and 10.5 mm. For other ranges of Z_v_, local changes in a_γ′_ of 0.004 Å, 0.002 Å and 0.006 Å were observed. The greatest changes of a_γ′_ were for the starting values of Z_v_ ranging between 1 mm and 1.5 mm, and for ending values of Z_v_, ranging between 10.5 mm and 12 mm. There is an a_γ′_ change with the value of 0.001 Å on the CH level. It is difficult to determine whether this change is related to the change of the growth conditions in the chamfer of the root or has a stochastic character. If such a small change results from the chamfer, then it is probably related to a significantly long distance (distance S_1_, Figure 1b) of the measuring line v from the chamfer of the root. Above the RA level, for Z_v_ equal to 8.5 mm, there is an increase in a_γ′_ of 0.002 Å.

In Figure 5b, by visualizing the α(Z_v_) relationship, generally, a continuous decrease in the α value can be observed for the entire measurement range of Z_v_. There are no extremes near the CH or RA levels. The large local decreases in the α value of 0.15° and 0.30° occur for the starting and ending Z_v_ values of the same Z_v_ ranges for which the a_γ′_ changes were greater. In the same ranges of Z_v_, large local increases in the β angle were also observed, but by significantly higher values (Δβ = 2.0°) in comparison to α changes (Δα = 0.15° or 0.30°). For the entire measuring range of Z_v_, the total β change is equal to 4.3° and α change is only equal to 0.65°. These values are marked in Figure 5b,c as Δβ_tot_ and Δα_tot_, respectively. No changes in β were observed for the CH level, while near the RA level, there was a very faintly distinct maximum. It is obvious that not only the presence of the local extremes of α(Z_v_) and/or β(Z_v_) relations demonstrate local dendrite bending. Also, the presence of local decreases or increases of α and/or β values, similar to those found in Figure 5 at the start and end of the Z_v_ measurement range, demonstrate such bends. However, the degree of these local bends is much smaller. From Figure 5, it can be concluded that the a_γ′_ changes at the beginning of line v and at the end of line v are related to dendrite bending manifested by changes in the α and β angles. However, it cannot be unequivocally concluded that dendrite bends cause a_γ′_ changes, as is the case for changes at the CH and RA levels of the blade region located near the CE [5].

The complex nature of changes in the a_γ′_(Z_v_) and lack of clear correlation of changes in its value on the CH and RA levels with changes of α and β is probably due to the presence of many macroscopic low-angle boundaries and the predominance of effects caused by the segregation of alloying element near these boundaries compared to the effects of the segregation of alloying elements close to the CH and RA levels due to dendrite bending. In addition, the trends of α decreasing and β increasing for the entire Z_v_ range may be related to the “fanning effect”, the consequences of which are intensified in the regions of the R-section located far from the CE (see Figure 1b), which masks local changes of α and β angles related to CH and RA levels.

**Figure 6:**
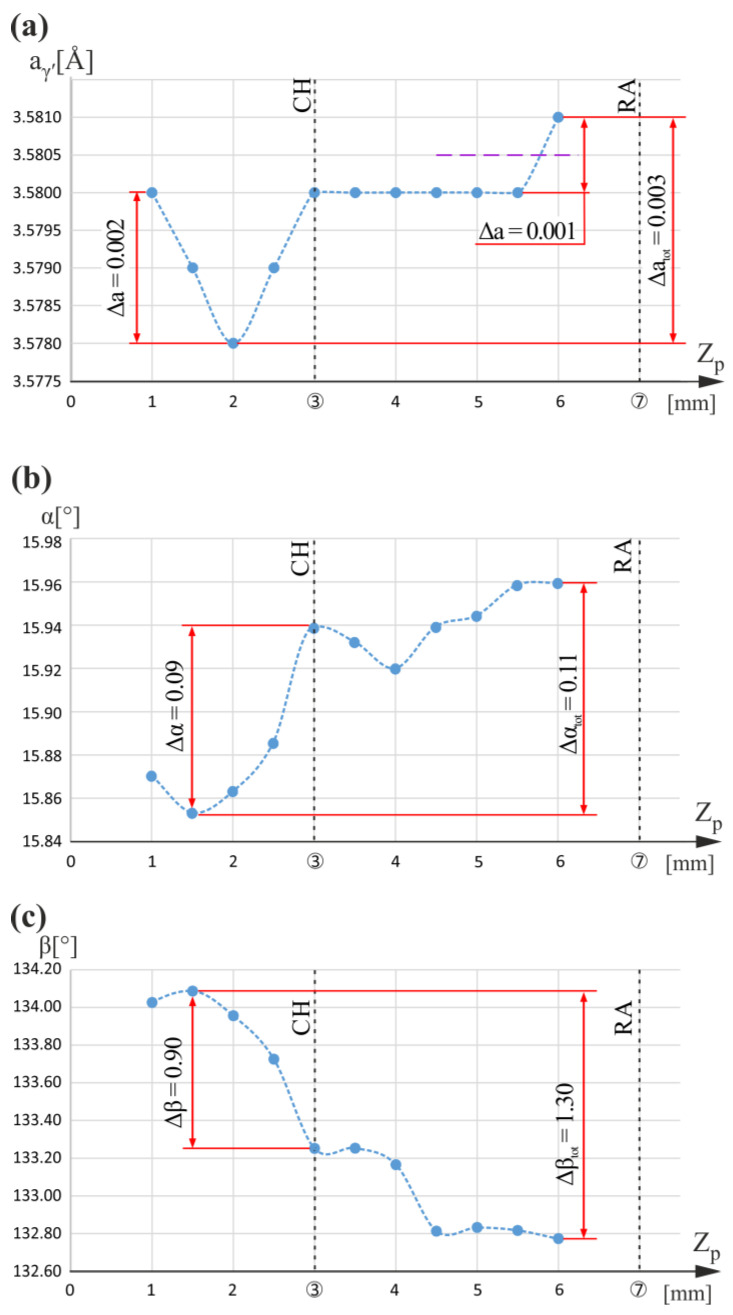
Graphs of the a_γ′_(Z_p_) (**a**), α(Z_p_) (**b**), and β(Z_p_) (**c**) relationships obtained for measuring line p. Z_p_ is parallel to Z_0_.

Figure 6 shows the graphs of the a_γ′_(Z_p_), α(Z_p_), and β(Z_p_) relationships obtained for measuring line p only located in the blade root (Figure 1a,b). For line p, there was no airfoil above the root fragment, and the line was simultaneously located near the root chamfer and root–airfoil connection (Figure 1b). Below the CH level, a local minimum at Z_p_ equal to 2 mm can be observed.

Figure 6b shows that for Z_p_ equal to 3 mm corresponding to the CH level, there is a faint maximum of the α(Z_p_) relationship. However, for the entire Z_p_ measuring range, the increasing trend, in general, is visible, whereas, for the β(Z_p_) relationship (Figure 6c), a decreasing trend is visible with some disturbance near the CH level. The rates of the α and β changes under the CH level (Z_p_ < 3 mm) are significantly higher compared to the areas localized above it. In addition, all changes in the β angle below and above the CH level are much greater than changes in the α angle. This means that the dendrites mostly rotate about the Z_p_ axis and tilt to a lesser extent.

For both L- and R-sections of the entire blade, measurements were made along four lines traced on the root areas over which the airfoil is not located. These measuring lines are p and k of the R-section and r_1_ and r_2_ of the L-section. Local changes in a_γ′_ values of measuring line p and their correlations with α changes have a different character than those occurring for measuring line k. Both lines are located near the chamfer of the root, but line p is distanced from the cooling bores, and the k line is located near the middle cooling bore (CB2). The following can be concluded by comparing the relation of the a_γ′_ lattice parameter on the coordinates of the measuring lines p and k. In the areas of root over which the airfoil is not located and which are distant from cooling bores (line p), the local changes in lattice parameter a_γ′_ related to the chamfer of the root are much lower than the changes in areas located closer to the cooling bores (line k). It is also confirmed by the measurement for lines b_1_ and b_2_ of the L-section, obtained in the first part of our research [5]. The a_γ′_ changes for these lines are only 0.002 Å and are much smaller than the a_γ′_ changes for line k. This means that the presence of the cooling bores significantly increased the heterogeneity of the alloying elements’ distribution in these root areas.

In general, the character and range of the a_γ′_, α, and β changes related to the CH level observed on all measuring lines of the R-section are complex, more complicated than those observed on the lines of the L-section. The reason may be the overlap of different effects, such as the “fanning effect” [35], and effects related to the existence of macroscopic low-angle boundaries occurring in the R-section area [35]. Therefore, the analysis of the influence of blade geometry on the lattice parameter of the γ′ phase and the primary orientation is difficult. The value of the α angle for all measuring lines of the R-section roughly ranges from 14.5° to 16.0° and is about twice as large for the areas of the L-section, where such value ranges roughly from 7.0° to 7.4°. Taking into account that the areas which belong to the R-section are located farther from the CE, it can be concluded that increasing the distance from the CE may be one of the reasons for the increase in the deviation of the [001]-type direction from the main blade axis. Comparing the a_γ′_ values for the entire R-section and L-section is difficult because the ranges of changes for each section are not separable, as is the case with the α angle value. For the entire R-section, a_γ′_ varies from 3.576 Å (minimum for line k) to 3.587 Å (maximum for line d_2_). On the other hand, for the entire L-section, a_γ′_ varies in the range from 3.578 Å (minimum for line t) to 3.582 Å (maximum for line b_1_)—which is also seen in [5].

For the areas of the L-section over which the airfoil is not located, the a_γ′_ values ranged from 3.579 Å to 3.581 Å (for both lines r_1_ and r_2_), as presented in [5]. On the other hand, for the mentioned areas of the R-section, the a_γ′_ value ranged in a very similar range from 3.578 Å to 3.581 Å for line p and in a much wider range from 3.576 Å to 3.582 Å for line k. Measuring lines r_1_, r_2_, and p are located far from the cooling bore, but the distance from the CE for line p is much longer than for r_1_ and r_2_. Comparing the ranges of changes in a_γ′_ for lines r_1_, r_2_, and line p leads to the conclusion that the distance from the CE does not play a significant role in these areas. On the other hand, comparing the ranges of changes in a_γ′_ for lines p and k, it can be concluded that for line k, the lower limit of a_γ′_ changes is decreased by 0.002 Å, and the upper limit is increased only by 0.001 Å. As a result, it can be deduced that the value of a_γ′_ is decreased for line k. Since line p is located far from any cooling bores, and line k is close to the second cooling bore (CB2), it can be additionally concluded that for the areas of root over which the airfoil is not located, the vicinity of the cooling bores causes the a_γ′_ value to decrease. This was also stated in [35]. According to the interpretation presented in [34], this means that there was an increase in the concentration of alloying elements, such as Re, W, Mo, and/or a decrease in Al, Ti, and Ta concentration.

**Figure 7:**
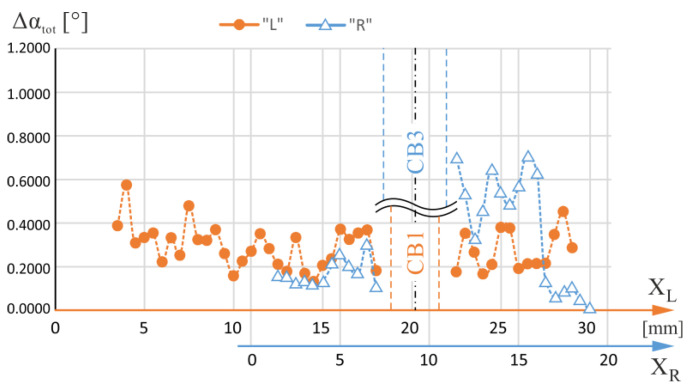
The total changes of α angle—Δα_tot_—defined for each measuring line with coordinates X_L_ of the L-section axis and X_R_ of the R-section axis. The lines were traced to pass through the blade area for which the airfoil is a continuation of the root. The X_L_ and X_R_ axes have been mutually shifted so that the geometric axes of the CB1 and CB3 overlap each other.

**Figure 8:**
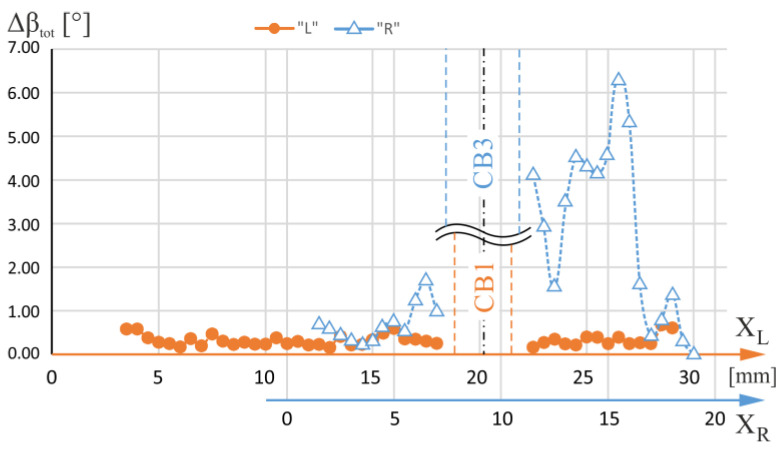
The total changes of β angle—Δβ_tot_—defined for each measuring line with coordinates X_L_ of the L-section axis and X_R_ of the R-section axis. The lines were traced to pass through the blade area for which the airfoil is a continuation of the root. The X_L_ and X_R_ axes have been mutually shifted so that the geometric axes of the CB1 and CB3 overlap each other.

Figure 7 shows the total ranges of the α angle changes—Δα_tot_—specified for each measuring line with X_L_ coordinates of the axis located on the L-section and X_R_ coordinates of the axis located on the R-section. The lines were traced to pass through the blade area for which the airfoil is a continuation of the root and therefore covered both the root and the airfoil. The X_L_ and X_R_ measurement axes in Figure 7 have been mutually shifted so that the geometric axes of the CB1 and CB3 overlap each other. Figure 8 shows the total changes of the β angle—Δβ_tot_—specified for the same L- and R-sections axes. From the analysis of Figure 7 and Figure 8, it can be concluded that, in general, for the R-section, on the right side of the CB3, the values of both the α and β total changes are higher than on the left side of the CB3. The area on the right side of the CB3 is separated from the region of the CE (Figure 1b) by the side surfaces of the CB3, while the area on the left side is not. This effect does not occur for CB1.

Significant α and β changes indicate the existence of macroscopic low-angle boundaries (LABs). They may be inherited by the root and then by the blade’s airfoil from the UDG layer (Figure 1a) of unsteady growth of dendrites [34]. The creation of the LABs in the UDG layer occurs at the initial crystallization stage of the root and is related to the disturbances in the lateral growth of dendrites arms from the region of the CE. Such disturbances are significantly increased when the walls of the CB3 are situated in the path of the laterally growing dendrites. From Figure 7 and Figure 8, it can be concluded that on the right and left sides of CB1, such changes in alpha and beta angles are comparable, which may be related to the small distance of CB1 from the region of the CE.

**Figure 9:**
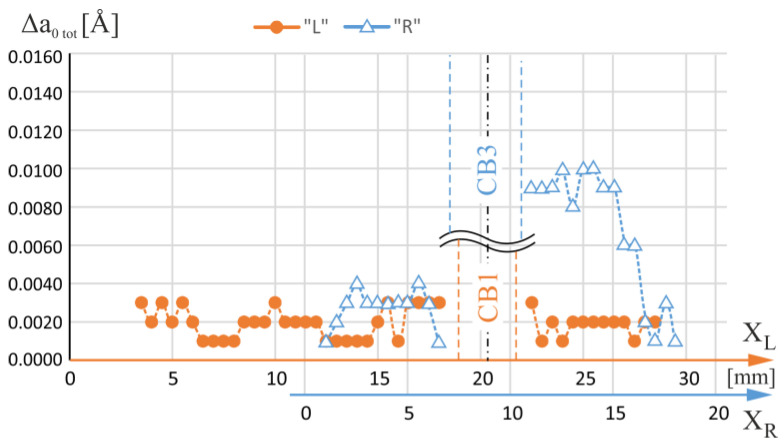
The total changes of lattice parameter a_γ′_ changes—Δa_tot_—defined for each measuring line with coordinates X_L_ of the L-section axis and X_R_ of the R-section axis. The lines were traced to pass through the blade area for which the airfoil is a continuation of the root. The X_L_ and X_R_ axes have been mutually shifted so that the geometric axes of the CB1 and CB3 overlap each other.

As shown in Figure 9, the value of Δa_tot_ for the R-section is on average higher than that of the L-section on the right side of the CB3. This means that there was an increase in the segregation of alloying elements. On the right side of the CB3, the values of Δα_tot_ and Δβ_tot_ are high. This may indicate the presence of many LABs that are manifested by changes in the crystal orientation. They are also related to the change in the value of the lattice parameter a_γ′_ related to the segregation of alloying elements. This segregation increases the heterogeneity of the chemical composition of the blade and is unfavorable to its strength because it increases the heterogeneity of stress distribution during its operation. Reducing the heterogeneity of cored blades by heat treatment seems difficult due to the presence of cooling bores which inhibit the diffusion process on their side surfaces. The heterogeneity is affected by two factors: the distance from the region of the CE and the presence of cooling bore walls in the UDG layer. Therefore, reduction of this type of segregation can be realized by the symmetrical positioning of the selector to reduce the distance of particular blade areas from the selector and abandon the fabrication of the cooling channels in the UDG layer.

## 4. Conclusions

Three areas of blade geometry were considered. The first area covered the blade root only with no airfoil continuation above the root; the second area covered both the root and the airfoil where the airfoil was a continuation of the root; the third area was part of the latter but located near the side walls of the cooling bore. For these areas, the influence of the blade geometry on a_γ′_ lattice parameter and the α and β angles of the dendrites’ primary orientation was analyzed based on changes in a_γ′_, α and β at two levels of alterations in the shape of the blade cross-section—the level of the root chamfer (CH) and the level of root–airfoil (RA) connection.

For the R-section areas, both for which the airfoil was not located directly above the root and those for which the airfoil was a continuation of the root, the correlation between the change in the lattice parameter a_γ′_ and extremes of the α angle describing the dendrite bending on or near the levels of CH and RA of the blade was less clear than in the case of the L-section. This is because macroscopic low-angle boundaries (LABs) are present for the R-section, and the consequences of the “fanning effect” are intensified. Therefore, the relationship details between the a_γ′_ changes and dendrite bending are difficult to study in the R-section located far from the selector’s continuer extension (CE), unlike in the L-section located closer to them.

For the areas of the blade in which the airfoil is a continuation of the root and which are located near the cooling bores, the total ranges of the changes in the α and β angles of the primary crystal orientation and lattice parameter of the γ′-phase are higher for the regions that are separated from the CE by surfaces of the cooling bores, unlike the regions of L-section close to the CE.

The change in the α angle near both the CH and RA levels appears together with the changes in the β angle that describes the rotation of the [001] direction about the main axis of the blade. For the R-section, far from the CE, the β changes are generally greater, which indicates the higher rotation of the dendrites compared to their tilting during growth.

In the areas of root over which the airfoil was not located and which were close to the cooling bores, the bends of the dendrites and changes in the lattice parameter a_γ′_ related to the chamfer of the root were much greater than the changes in regions of the root distanced from the bores.

Near the levels of the R-section, at which significant changes of the α and β angles occurred, there were local changes in the a_γ′_ ranging from 0.002 Å to 0.006 Å. The upper limit of the range of the changes was much larger than for the same L-section levels, which were only 0.002 Å.

Increasing the distance from the continuer extension (CE) may be one reason for the increase in the changes and value of the α angle that describes the deviation of the [001]-type direction from the main blade axis.

## Data Availability

Not applicable.
